# Inhibiting 4E-BP1 re-activation represses podocyte cell cycle re-entry and apoptosis induced by adriamycin

**DOI:** 10.1038/s41419-019-1480-x

**Published:** 2019-03-11

**Authors:** Fang Li, Xing Mao, Qiyuan Zhuang, Zhonghua Zhao, Zhigang Zhang, Huijuan Wu

**Affiliations:** Department of Pathology, School of Basic Medical Sciences, Fudan University, Shanghai, China

## Abstract

Podocyte loss is one of the determining factors for the progression toward glomerulosclerosis. Podocyte is terminally differentiated and does not typically proliferate following injury and loss. However, recent evidence suggested that during renal injury, podocyte could re-enter the cell cycle, sensitizing the cells to injury and death, but the molecular mechanisms underlying it, as well as the cell fate determination still remained unclear. Here, using *NPHS2 Cre; mT/mG* transgenic mice and primary podocytes isolated from the mice, we investigated the effect of mammalian target of rapamycin complex 1 (mTORC1)/4E-binding protein 1 (4E-BP1) signaling pathway on cell cycle re-entry and apoptosis of podocyte induced by adriamycin. It was found that podocyte cell cycle re-entry could be induced by adriamycin as early as the 1st week in vivo and the 2nd hour in vitro, accompanied with 4E-BP1 activation and was followed by podocyte loss or apoptosis from the 4th week in vivo or the 4th hour in vitro. Importantly, targeting 4E-BP1 activation by the RNA interference of 4E-BP1 or pharmacologic rapamycin (inhibitor of mTORC1, blocking mTORC1-dependent phosphorylation of its substrate 4E-BP1) treatment was able to inhibit the increases of PCNA, Ki67, and the S-phase fraction of cell cycle in primary podocyte during 2–6 h of adriamycin treatment, and also attenuated the following apoptotic cell death of podocyte detected from the 4th hour, suggesting that 4E-BP1 could be a regulator to manipulate the amount of cell cycle re-entry provided by differentiated podocyte, and thus regulate the degree of podocyte apoptosis, bringing us a new potential podocyte-protective substance that can be used for therapy.

## Introduction

Glomerulosclerosis is the major pathological process leading to end-stage renal disease^1^. Depletion of podocyte, which is the critical constituent of the glomerular filtration barrier, is crucial for the progression of glomerular disorders toward glomerulosclerosis^2^. Terminally differentiated podocytes are highly specialized cells that do not typically proliferate in response to injury. However, forced re-entry of terminally differentiated podocytes into the cell cycle is possible, as was reported in human glomerular diseases including collapsing glomerulopathy, IgA nephropathy, focal segmental glomerulosclerosis (FSGS), and lupus nephritis^3–6^. This has also been demonstrated in some animal experimental models including the passive Heymann nephritis (PHN) model of membranous nephropathy, anti-Thy 1.1 nephritis, and 5/6-nephrectomy^7–11^, and illustrated by a number of experimental manipulations including viral infections, overexpression of cyclin D1 and CDK 4/6^12–14^, ectopic expression of the Notch intracellular domain^15^ and elongation factor 2^16^.

However, the consequences of podocyte cell cycle re-entry are dramatic^17^. Most findings support the concept that podocyte cell cycle re-entry represents a stressful event that drives podocyte loss either by death, detachment, or both. Hara et al.^18^ revealed in FSGS and lupus nephritis patients that podocytes given a proliferative response were more susceptible to detachment and loss^10,19^. Using a mouse-immortalized podocyte cell line, Hagen et al.^20^ proved that secondary injuries stimulated significantly increased cell loss in podocytes entering the cell cycle. This indicated that podocytes undergoing cell cycle re-entry, which exhibited biomarkers of cell cycle progression such as proliferating cell nuclear antigen (PCNA) or Ki67, were more vulnerable to injury and death. Accordingly, investigation of the driving mechanism behind podocyte cell cycle re-entry is important for preventing podocyte cell cycle progression and the subsequent suppression of podocyte injury in glomerular diseases.

The mammalian target of rapamycin complex 1 (mTORC1) signaling pathway, known as a primary pathway controlling cell proliferation and growth, is differentially activated in different podocyte stages^21,22^. During development, mTORC1 activity is upregulated in podocyte progenitors, but the hyper-activation of mTORC1 is downregulated as progenitors differentiate into podocytes^23^. mTORC1 regulates cell cycle progression and cell growth by modulating mRNA translation through the phosphorylation of its two downstream effectors: the ribosomal protein S6 kinase 1 and the eukaryotic translation initiation factor 4E-binding protein 1 (4E-BP1)^24–26^. Research has shown that the regulation of cell proliferation and size could be independent, S6Ks have a key role in the control of cell size, whereas 4E-BPs regulate cell proliferation through modulation of the cell cycle rather than cell size^27^.

Recent studies have revealed that upon mTOR-dependent phosphorylation of 4E-BP1, 4E-BP1 is released from eIF4E, allowing eIF4E to assemble with other translation initiation factors to initiate cap-dependent translation^28–31^. eIF4E is thought to increase the translation of transcripts possessing either complex 5′-untranslated region secondary structures and/or upstream open reading frames, which often encode proteins associated with a proliferative response^32,33^. Thus, hyper-activation of the mTORC1/4E-BP1 pathway involved in cell cycle progression is observed in progenitor podocytes, as soon as progenitors differentiate into podocytes, the enhanced phosphorylation of 4E-BP1 mediated by mTORC1 decreases enough to a low level only maintains normal podocyte function^23^. However, whether mTORC1/4E-BP1 could be re-activated and implicated in the cell cycle re-entry of terminally differentiated podocytes under stress remains unknown.

In this study, we reported that adriamycin stimulated a potent and limited cell cycle re-entry in mature podocytes, accompanied by hyper-phosphorylation of 4E-BP1. Furthermore, we found targeting 4E-BP1 activation with the RNA interference of 4E-BP1 or pharmacologic rapamycin (inhibitor of mTORC1, blocking mTORC1-dependent phosphorylation of its substrate 4E-BP1) treatment could inhibit podocyte cell cycle re-entry and suppress the podocyte injury and loss induced by adriamycin, providing a new potential target for ameliorating podocyte injury in glomerular disorders.

## Results

### Adriamycin stimulated podocyte cell cycle re-entry followed by podocyte injury and loss

**Fig. 1:**
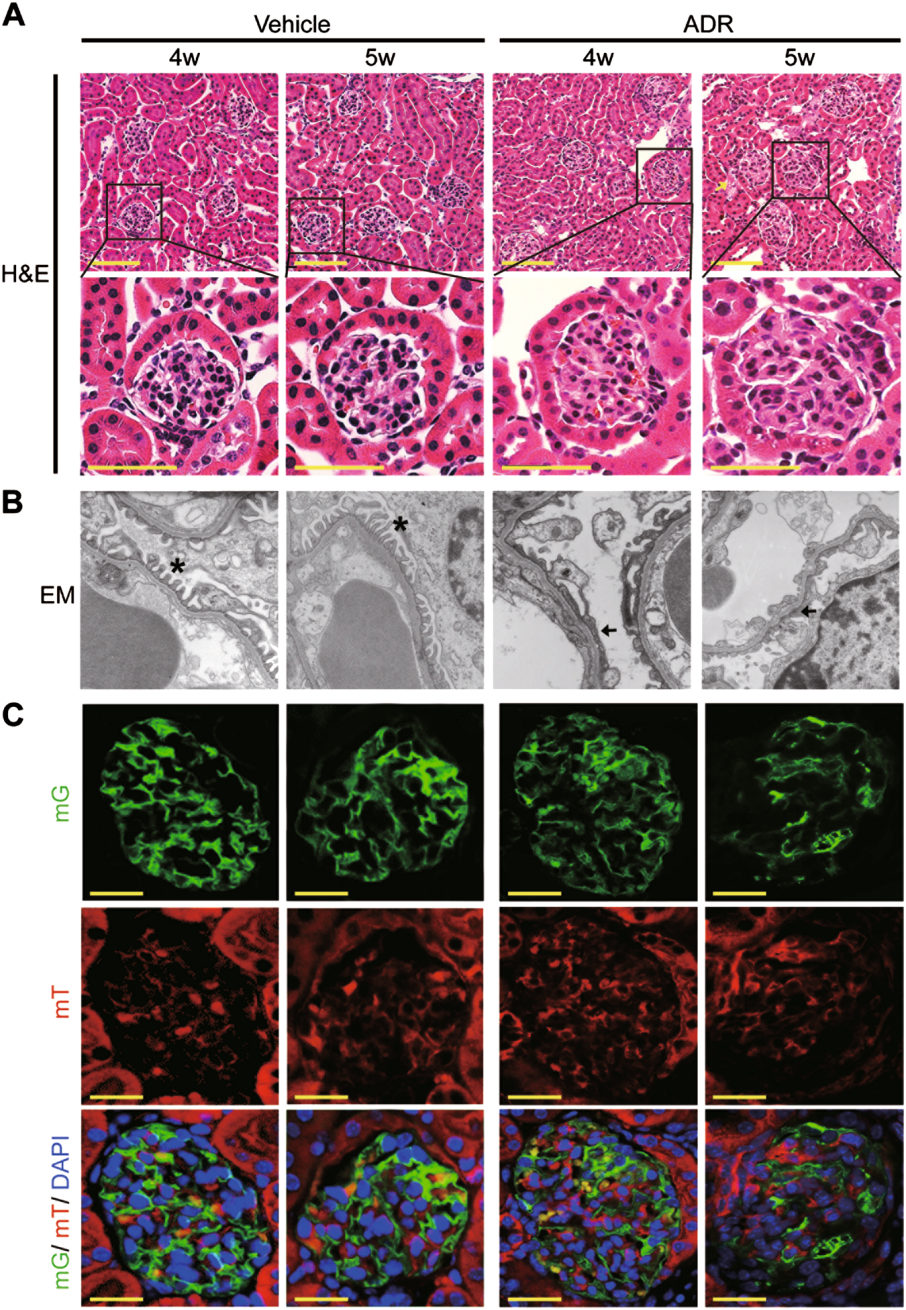
The morphology of glomeruli in *NPHS2 Cre; mT/mG* mice treated with adriamycin or vehicle A low dose of adriamycin, 15 mg/kg Body weight, was chosen to induce podocytes injury. Protein cast was detected at the 5th week after adriamycin treatment (yellow arrow), shown are representative kidney sections with H&E staining **a**. Images of electron microscopy from *NPHS2 Cre; mT/mG* mice treated with vehicle or adriamycin for 4 or 5 weeks (4 mice per group), podocyte foot process was normal in vehicle group (asterisk), whereas the wide podocyte foot process effacement were detected at 4 and 5 weeks after adriamycin treatment (arrow) **b**. **c** Paraffin-embedded kidney slides collected from *NPHS2 Cre; mT/mG* mice treated with adriamycin or vehicle were used for direct fluorescence microscopy after fast deparaffinization, showing fluorescence intensity of mG expressed by podocytes slightly was decreased at the 4th and 5th week after adriamycin treatment. (Scale bars: 100 μm in the upper row of **a**, 50 μm in the down row of **a**, 6000 × in **b**, and 25 μm in all images of **c**)

By crossing *mT/mG* mice (*Gt(ROSA)26Sor*^*tm4(ACTB-tdTomato,-EGFP)Luo*^) with transgenic mice expressing Cre recombinase under the control of the podocin (NPHS2) promoter, podocyte-specific membrane-localized enhanced-GFP transgenic mice (*NPHS2 Cre; mT/mG* mice) were used to trace podocyte change (Figure S1A). As shown in Figure S1B, podocytes were specifically membrane-labeled with green fluorescent protein (mG) and other cells were membrane-labeled with red fluorescent protein td-Tomato (mT). Adriamycin (15 mg/kg body weight) was used to induce the adriamycin-induced nephropathy, a classic model of glomerular disease. As shown in Fig. 1a, by the 5th week after adriamycin treatment, protein casts (yellow arrow) were found in adriamycin-treated mice, but no significant differences were observed in glomerular morphology between the adriamycin-treated and vehicle-treated mice. However, electron microscopy revealed a wide podocyte foot process effacement (Fig. 1b, black arrow) in adriamycin-treated mice in the 4th and 5th weeks following treatment. Moreover, using paraffin-embedded kidney slides collected from *NPHS2 Cre; mT/mG* mice for direct fluorescence microscopy after fast deparaffinization (Fig. 1c), we found that the density of green fluorescence representing podocytes decreased in the 4th and 5th weeks following adriamycin treatment, indicating podocyte injuries. Consistent with the progressive podocyte injuries, a statically significant increase in the urinary albumin/creatinine ratio was detected in the 4th and 5th weeks following treatment (Figure S2A). Sodium dodecyl sulphate–polyacrylamide gel electrophoresis (SDS-PAGE) and coomassie blue staining confirmed the increased urinary albumin excretion in the 4th week, which was then aggravated by the 5th week (Figure S2B). By the 5th week following adriamycin treatment, a statistic increase in the blood urea nitrogen (BUN) level was detected (Figure S2C). These data suggested the successful establishment of an adriamycin-induced nephropathy mouse model in which podocyte injury and loss were detected from the 4th week following adriamycin treatment.

**Fig. 2:**
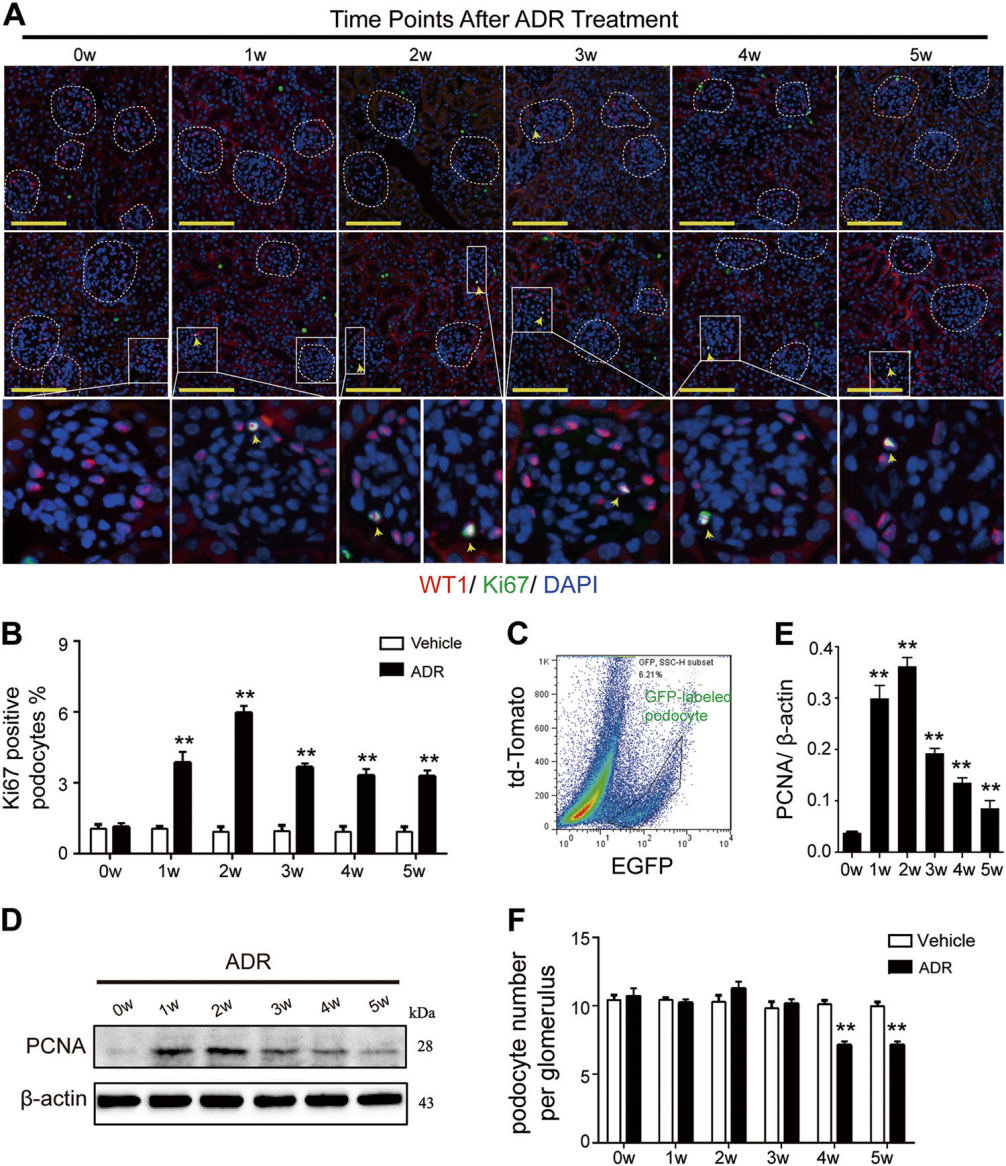
Adriamycin stimulated podocyte cell cycle re-entry since the 1st week after treatment **a** Double immunofluorescence staining with WT1 and Ki67 revealed more Ki67-positive podocyte (yellow arrow) in adriamycin-treated mice compared with the vehicle group. **b** The percentage of Ki67-positive podocyte was calculated from 40 randomly captured images for each group (10 images per mouse, 4 mice per group) at each time point after adriamycin treatment. Only WT1 and Ki67 double positive nuclei were counted for the numerator, which was divided by the sum of WT1-positive cells within captured images and then multiplied by 100%, indicating that adriamycin treatment induced an upregulated ratio of Ki67 and WT1 double-positive cells, which peaked at the 2nd week and then decreased from the 3rd week, but still remained higher compared with control group at the 5th week. **c** m-EGFP-labeled podocyte was isolated through fluorescence-activated cell sorting (FACS) and **d**, **e** then was lysed and subjected to immunoblotting analysis, revealing an upregulated PCNA expression level since the 1st week after adriamycin treatment. Shown were representative blots from at least three separate experiments with similar results, the statistic based on densitometric analysis of at least 3 (up to 6) independent experiments. **f** The number of WT1-positive cells was counted for the numerator from 40 randomly captured images for each group (same as above) at each time point, which was divided by the sum of glomeruli within captured images. The podocyte number per glomerulus was statistically decreased at the 4th and 5th week after adriamycin treatment. Data were presented as means ± SEM, **P < 0.01 for adriamycin-treated mice vs. vehicle-treated mice. (Scale bars: 100 μm)

To trace podocyte cell cycle changes in the adriamycin-induced nephropathy mouse model, double immunofluorescence staining with two antibodies, WT1 (marker for podocyte nuclei) and Ki67 (proliferating cell marker), was performed to examine Ki67 expression in podocytes. The Ki67-positive podocyte rate increased as early as the 1st week following adriamycin treatment, peaked at the 2nd week, and decreased from the 3rd week onwards, however, the rates remained higher than the control group until the 5th week (Fig. 2a, b). Double immunofluorescence staining with podocin (a cytoplasmic marker of differentiated podocytes) and Ki67 revealed the same trend (Figure S3) shown in Fig. 2a, b, confirming that the Ki67-positive cells were mature podocytes. Moreover, mG-labeled podocytes were isolated from the experimental mice through fluorescence-activated cell sorting (FACS) (Fig. 2c), and the protein expression of PCNA was detected using immunoblotting analysis (Fig. 2d, e). The PCNA expression level increased from the 1st week following adriamycin treatment, peaked by the 2nd week, then decreased but remained above the basal level from the 3rd to the 5th week, confirming podocyte cell cycle re-entry under adriamycin-induced toxicity. Interestingly, no increase in podocyte numbers were detected in the first three weeks when both Ki67 and PCNA expression were significantly elevated (Fig. 2f), whereas podocyte loss was detected in the 4th and 5th weeks after adriamycin treatment. This indicated that although adriamycin stimulated podocytes to re-enter the active phases of cell cycle soon after treatment, the podocytes might not really divide. As long as podocytes were suffering from adriamycin-induced toxicity, podocyte injury and loss occurred.

### 4E-BP1 was activated when podocytes underwent cell cycle re-entry

**Fig. 3:**
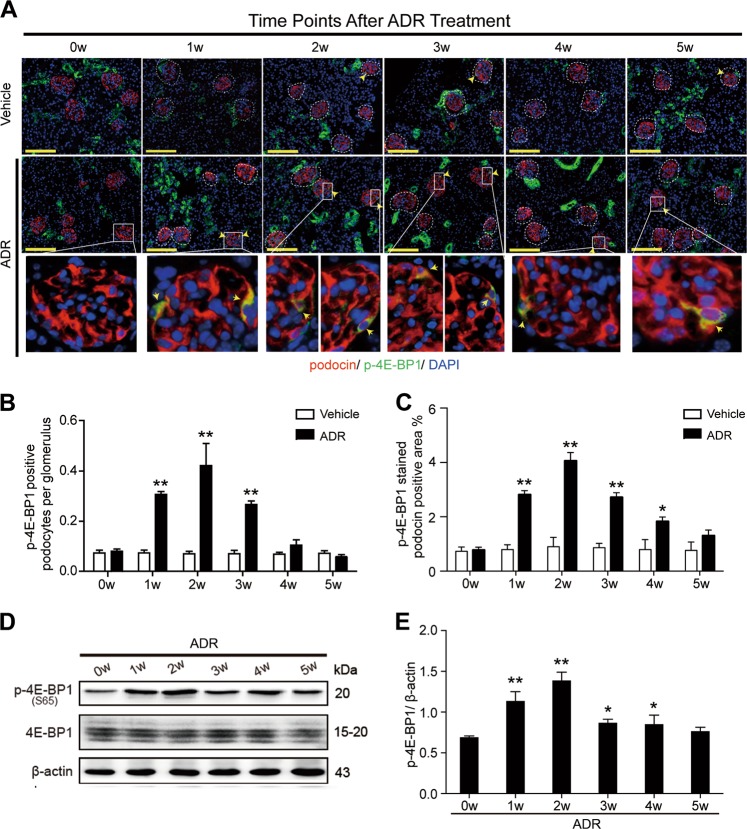
4E-BP1 phosphorylation was significantly enhanced in podocyte from the 1st week after adriamycin treatment **a** Double immunofluorescence staining with podocin and p-4E-BP1 (S37/46) in adriamycin or vehicle-treated mice. A dramatically enhanced phosphorylation of 4E-BP1 (S37/46) (yellow arrow) in podocyte of adriamycin-treated mice was detected. **b**, **c** The number of p-4E-BP1 (S37/46)-positive podocytes per glomerulus **b** and p-4E-BP1 (S37/46) stained podocin-positive area **c** were both calculated from 40 randomly captured images for each group (10 images per mouse, 4 mice per group) at each time point. p-4E-BP1 and podocin double-positive cells were counted for the numerator, which was divided by the sum of glomerular within captured images **b**, or p-4E-BP1-stained podocin-positive area was calculated for the numerator, which was divided by the sum of podocin stained glomerular area **c**. Both the number of p-4E-BP1-positive podocytes per glomerulus and the p-4E-BP1-stained podocin-positive area were markedly increased during the first 2 weeks and gradually decreased since the 3rd week after adriamycin treatment. Data were presented as means ± SEM, ^*^P < 0.05, ^**^P < 0.01 for adriamycin-treated mice vs. vehicle-treated mice. **d**, **e** Immunoblotting analysis of proteins from isolated podocytes indicated aberrantly upregulated phosphorylation of 4E-BP1(S65) during the first 5 weeks after adriamycin treatment. Shown were representative blots from at least three separate experiments with similar results, the statistic based on densitometric analysis of at least three (up to six) independent experiments. Data were presented as means ± SEM, ^*^P < 0.05, ^**^P < 0.01 for adriamycin-treated mice vs. vehicle-treated mice. (Scale bars: 100 μm)

4E-BP1 is an important downstream target of mTORC1 phosphorylation and regulates mTORC1-mediated cell proliferation rather than cell size. Once activated, phosphorylated 4E-BP1 selectively promotes the translation of messenger RNAs that encode proliferation-promoting proteins and proteins involved in cell cycle progression to positively regulate cell cycle progression. Upregulated mTORC1/4E-BP1 activity is detected in progenitor podocytes, whereas in mature podocytes maintaining homeostasis, mTORC1/4E-BP1 activity remains at a low level to maintain normal podocyte function. In this study, podocyte cell cycle re-entry induced by adriamycin was detected (Fig. 2), we thus wondered whether 4E-BP1 was re-activated. Using double immunofluorescence staining with p-4E-BP1 (S37/46) and podocin (Fig. 3a–c), or using immunoblotting to analyze 4E-BP1 activity in FACS-isolated podocyte (Fig. 3d, e), we investigated 4E-BP1 activation in podocytes of experimental mice and found very similar results. Significantly enhanced 4E-BP1 phosphorylation was observed in podocyte from the 1st week following adriamycin treatment when cell cycle re-entry was detected. The phosphorylated 4E-BP1 expression level continued to increase and peaked at the 2nd week, then gradually decreased from the 3rd week of adriamycin treatment, displaying the trend consistent with Ki67 and PCNA, indicating that p-4E-BP1 might play a role in mediating the cell cycle change of adriamycin-stimulated podocytes.

### Podocyte cell cycle re-entry was mediated by 4E-BP1 activation

Glomeruli from *NPHS2 Cre; mT/mG* mice were isolated and cultured (Figure S4A). When cells grew out of the glomeruli, mG-labeled podocytes were isolated by FACS (Figure S4B) and identified through immunofluorescence staining with podocyte markers WT1 and nephrin (Figure S4C& D).

**Fig. 4:**
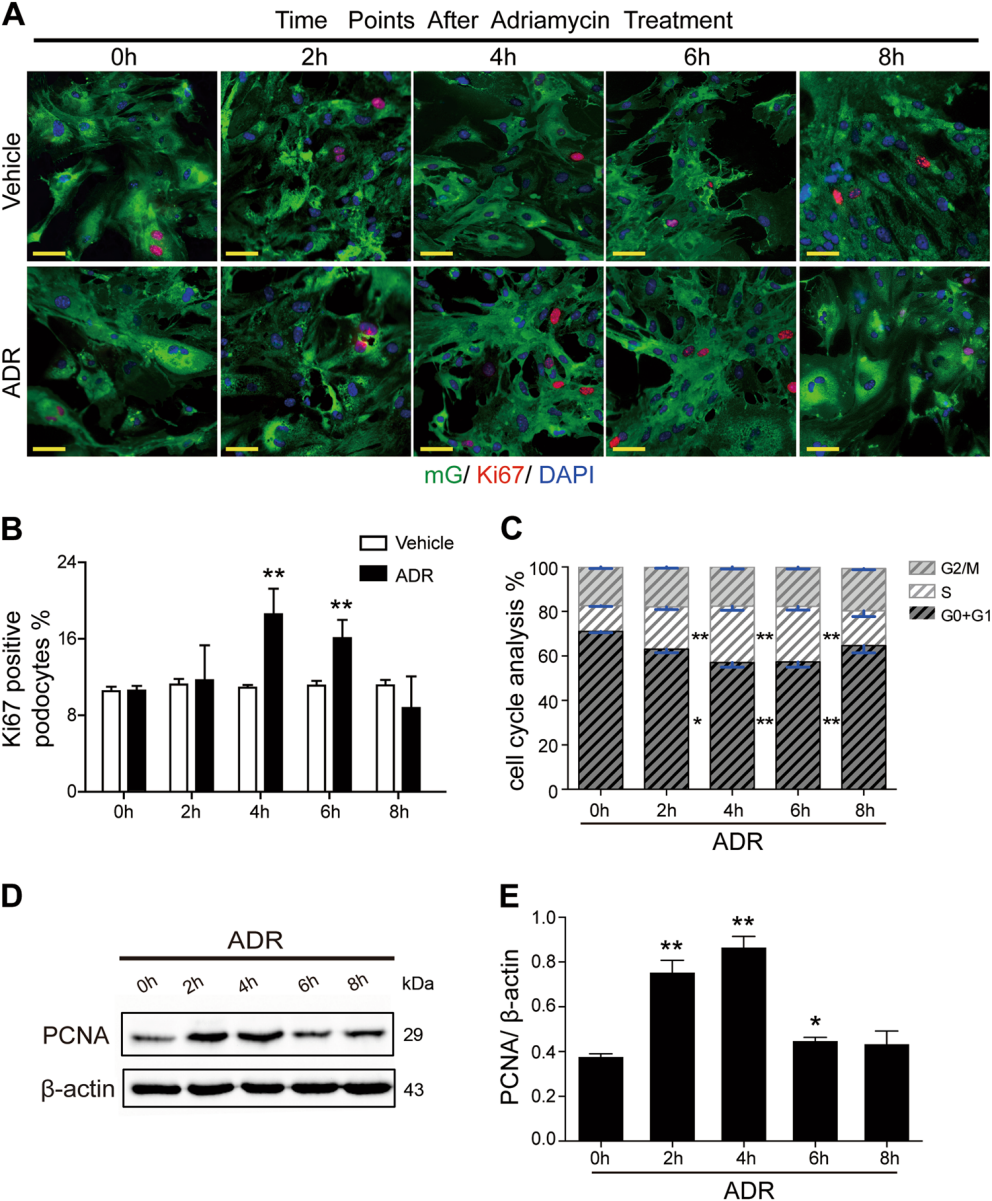
Adriamycin induced cell cycle re-entry of primary podocyte **a** mG-labeled primary podocytes were treated with adriamycin (1 mg/L) for 2, 4, 6, or 8 h and then stained with Ki67 and DAPI. vehicle (PBS)-treated group was used as control. **b** The percentage of Ki67-positive podocyte was calculated from 45 randomly captured images for each group (15 images per slide, three slides per group) at each time point. Only Ki67-positive nucleus were counted for the numerator, which was divided by the sum of DAPI-positive nuclei and then multiplied by 100%, showing Ki67-positive podocyte rate was increased at 4 and 6 h after adriamycin treatment. Data were shown as means ± SEM, **P < 0.01 for adriamycin-treated podocytes vs. vehicle-treated podocytes. **c** Flow cytometry analysis of cell cycle in podocytes. Podocytes were incubated with 1 mg/L adriamycin for 0, 2, 4, 6, and 8 h before were analyzed using flow cytometry to assess cell cycle. For each sample, 10000 single, GFP-positive events were analyzed. Data represented the mean ± SEM of at least three independent experiments. ^*^P < 0.05, **P < 0.01 for adriamycin-treated podocytes vs. vehicle-treated podocytes. **d**, **e** Immunoblotting showed markedly increased PCNA expression at 2, 4 and 6 h after adriamycin treatment. Representative blots shown were from at least three separate experiments with similar results, and the statistic based on densitometric analysis of at least three (up to six) independent experiments. Data were shown as means ± SEM, **P < 0.01 for adriamycin-treated podocytes vs. vehicle-treated podocytes. (Scale bars: 50μm)

We then cultured the pure mG-labeled podocytes and treated them with adriamycin (1 mg/L) or control solution (phosphate-buffered saline; PBS) for 2, 4, 6, and 8 h. As shown in Fig. 4, podocytes presented cell cycle re-entry after the 2nd hour of adriamycin treatment, with increased Ki67-positive rates (Fig. 4a, b) and upregulated PCNA expression levels (Fig. 4d, e). Notably, the Ki67-positive rates as well as the PCNA expression levels of primary podocytes peaked in the 4th hour, but decreased from the 6th hour of adriamycin treatment. Cell cycle analysis demonstrated the similar results, the percentage of podocyte in the S-phase was statistically increased after 2 h of adriamycin treatment, peaked in the 4th hour, and decreased after the 6th hour of treatment (11.59% 19%, 25.3%, 25% and 15.6% at 0, 2, 4, 6, and 8 h, respectively) (Fig. 4c). All these findings indicated that under adriamycin stimulation, mature podocytes could re-enter into the cell cycle, but this cell cycle progression was limited.

**Fig. 5:**
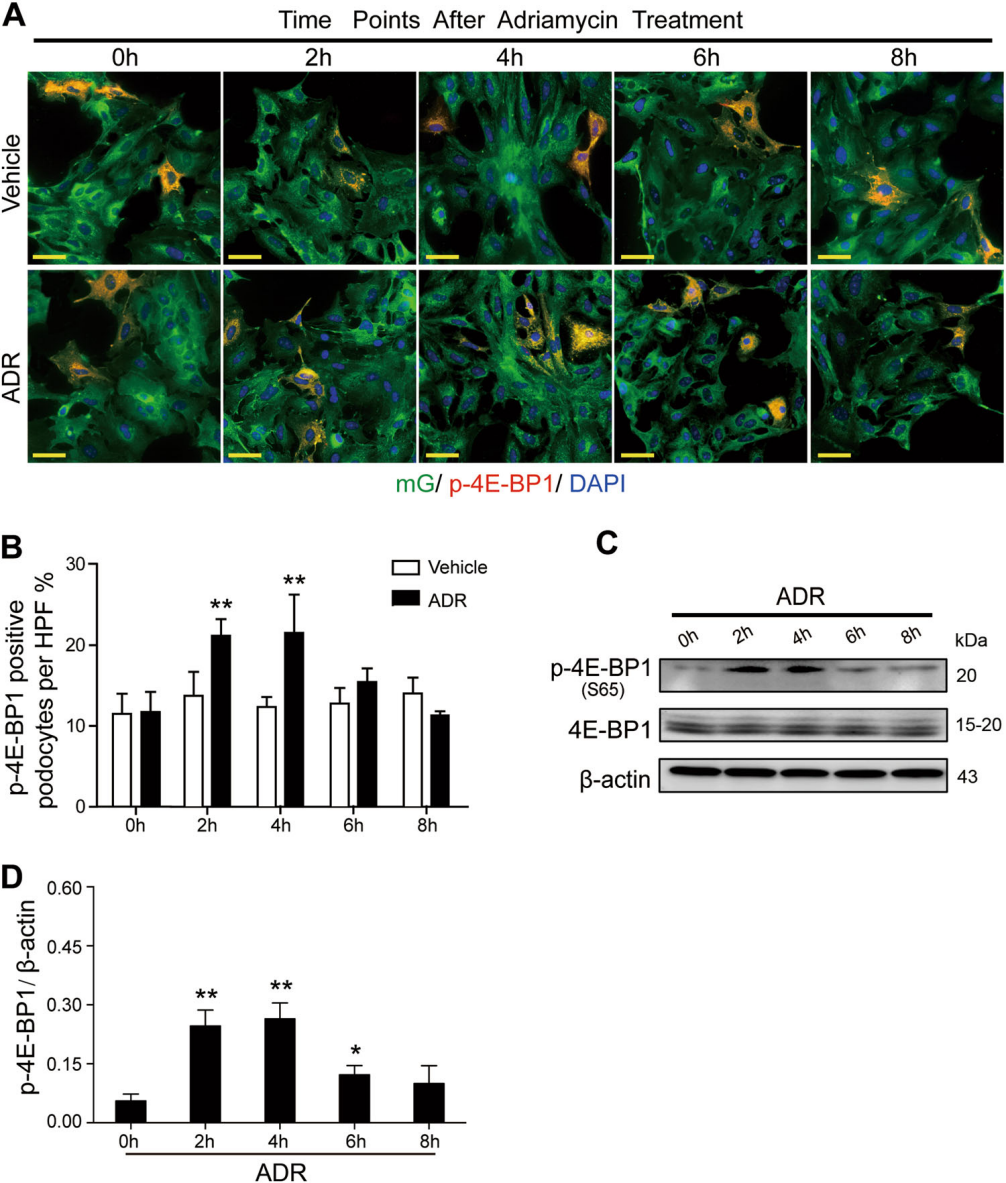
4E-BP1 phosphorylation was enhanced in primary podocytes treated with adriamycin **a** The mG-labeled primary podocytes, stained with p-4E-BP1 (S37/46) and DAPI, were observed by immunofluorence microscope after adriamycin (1 mg/L) treatment (0, 2, 4, 6, and 8 h, respectively). **b** The percentage of p-4E-BP1-positive podocytes was calculated from 45 randomly captured images for each group (15 images per slide, three slides per group) at each time point. Phosphorylated 4E-BP1 positive podocytes were counted for the numerator, which was divided by the sum of DAPI-positive podocytes and then multiplied by 100%. Data were shown as means ± SEM, **P < 0.01 for adriamycin-treated podocytes vs. vehicle-treated podocytes. **c**, **d** Immunoblotting of proteins from primary podocytes treated with adriamycin showed an upregulated 4E-BP1 phosphorylation at 2, 4, and 6 h after adriamycin treatment. Representative blots shown were from at least three separate experiments with similar results, the statistic based on densitometric analysis of at least three (up to six) independent experiments. Data were shown as means ± SEM, ^*^P < 0.05, ^**^P < 0.01 for adriamycin-treated podocytes vs. vehicle-treated podocytes (Scale bars: 50 μm)

Moreover, using immunofluorescence staining and immunoblotting analysis, we found the phosphorylation of 4E-BP1 was enhanced following the 2nd hour of adriamycin treatment, peaked at the 4th hour, and then decreased from the 6th hour onward (Fig. 5a–d). This displayed the same trend as proliferative indicators Ki67 and PCNA, suggesting the activation of 4E-BP1 might have a role in the cell cycle re-entry.

**Fig. 6:**
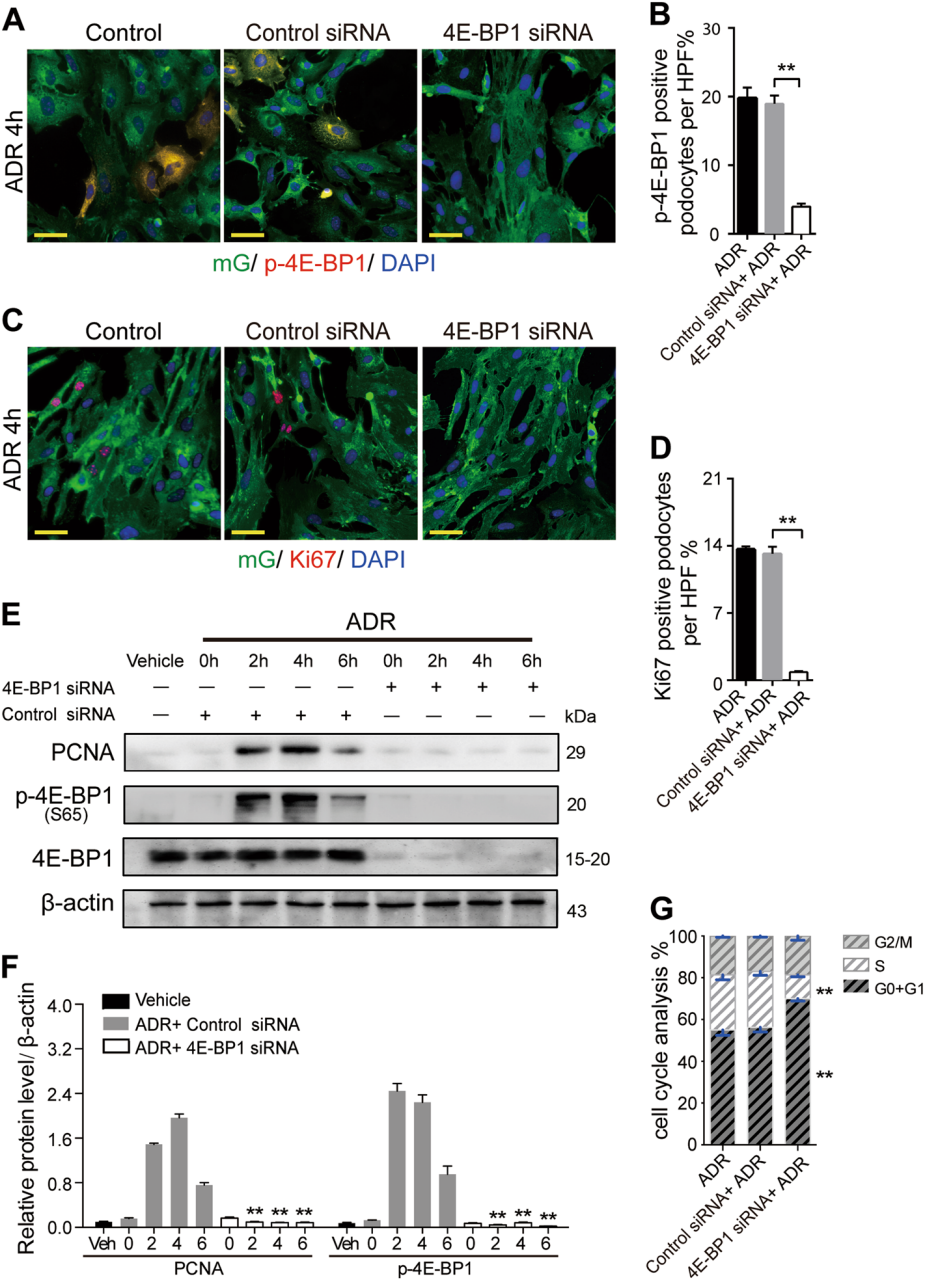
Knocking-down 4E-BP1 inhibited primary podocyte cell cycle re-entry **a**–**d** Primary podocytes membrane-labeled with EGFP were interfered by 4E-BP1 siRNA (25 nM) or control siRNA (25 nm) for 48 h, and then treated with adriamycin (1 mg/L) for 4 h, which was followed by the immunofluorescence staining with p-4E-BP1 (S37/46) or Ki67 immediately. Phosphorylated 4E-BP1 or Ki67-positive podocytes were calculated from 45 randomly captured images for each group (15 images per slide, three slides per group). p-4E-BP1 or Ki67-positive cells were counted for the numerator, which was divided by the sum of DAPI-positive cells and then multiplied by 100%. Data were shown as means ± SEM, **P < 0.01 for 4E-BP1 siRNA vs. control siRNA. **e**, **f** Immunoblotting results showed that inhibition of 4E-BP1 blunted both adriamycin-induced 4E-BP1 phosphorylation and PCNA expression. Representative blots were from at least three separate experiments with similar results, the statistic based on densitometric analysis of at least three independent experiments. Data were shown as means ± SEM, ^**^P < 0.01 for control siRNA vs. 4E-BP1 siRNA. **g** Cell cycle analysis of podocytes incubated with adriamycin for 4 h, with or without pre-treated with 4E-BP1 siRNA. Data were shown as means ± SEM, **P < 0.01 for 4E-BP1 siRNA vs. control siRNA. (Scale bars: 50 μm)

To confirm whether this 4E-BP1 activation was directly involved in mediating podocyte cell cycle re-entry, primary podocytes were exposed to 4E-BP1 siRNA for 48 h before adriamycin treatment, or pharmacologically treated with rapamycin (10 nm), in the presence of adriamycin, to inhibit phosphorylated 4E-BP1 expression in podocyte. Using immunofluorescence staining, the Ki67-positive podocyte rate was shown to increase, peaking in the 4th hour of adriamycin treatment, and was significantly attenuated by 4E-BP1 RNA interference (Fig. 6a–d). Using immunoblotting analysis, elevated PCNA expression levels were detected in podocytes from the 2nd to the 6th hour of adriamycin treatment and were markedly suppressed by the transient siRNA-based 4E-BP1 knockdown (Fig. 6e, f) or rapamycin treatment (Fig. 8a, b). Using the cell cycle analysis, the podocyte cell cycle progression, showing as an increase of S-phase fraction following 2–6 h of adriamycin treatment, was dramatically diminished by RNA interference-mediated knockdown of 4E-BP1 (Fig. 6g) or rapamycin treatment (decreased to 12.3, 12, 11, and 12% at 2, 4, 6, and 8 h respectively) (Fig. 8c), demonstrating that the 4E-BP1 activation played a key role in this cell cycle progression of primary podocytes.

### 4E-BP1 activity suppression attenuated adriamycin-induced podocyte apoptosis

**Fig. 7:**
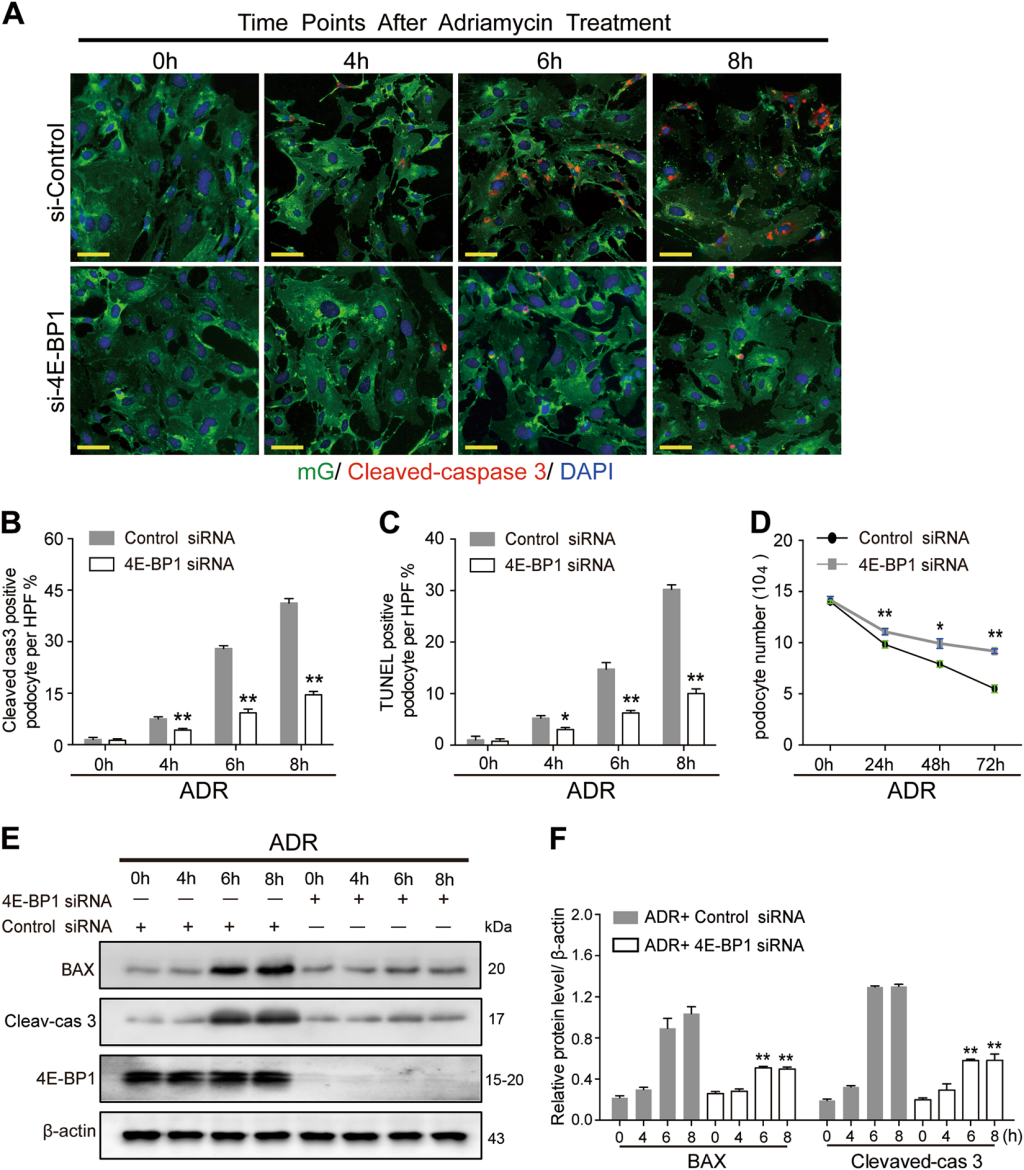
Downregulating 4E-BP1 decreased adriamycin-induced apoptotic cell death of podocytes **a**, **b** Downregulating 4E-BP1 through 4E-BP1 siRNA prevented caspase-3 activation in adriamycin (1 mg/L) treated primary podocytes. Immunofluorescence staining of cleaved-caspases-3 (red) and nuclear DAPI (blue) in primary podocytes treated with adriamycin. Cleaved-caspases-3 positive podocytes were scored from 45 randomly captured images for each group (15 images per slide, three slides per group), in both control siRNA treated and 4E-BP1 siRNA-treated groups. Data were shown as means ± SEM, ^**^P < 0.01 for 4E-BP1 siRNA vs. control siRNA. **c** Cell apoptosis analysis using DAPI and TUNEL staining. TUNEL-positive podocytes were scored in both control siRNA-treated and 4E-BP1 siRNA-treated groups (15 images per slide, three slides per group). Data were shown as means ± SEM, *P < 0.05, ^**^P < 0.01 for 4E-BP1 siRNA vs. control siRNA. **d** Podocyte number decreased among groups of primary podocyte in presence of adriamycin treatment for 24, 48, or 72 h. The loss of podocyte number caused by adriamycin was partially suppressed by 4E-BP1 siRNA treatment. Data were shown as means ± SEM, *P < 0.05, ^**^P < 0.01 for 4E-BP1 siRNA vs. control siRNA. **e,** **f** Immunoblotting analysis of BAX, cleaved-caspases-3, and 4E-BP1 in control siRNA or 4E-BP1 siRNA treated podocytes in presence of adriamycin treatment for 4, 6, or 8 h. Shown were one from at least three separate experiments with similar results. The statistic based on densitometric analysis of at least three independent experiments. Data were shown as means ± SEM, *P < 0.05, ^**^P < 0.01 for 4E-BP1 siRNA vs. control siRNA. (Scale bars: 50 μm)

**Fig. 8:**
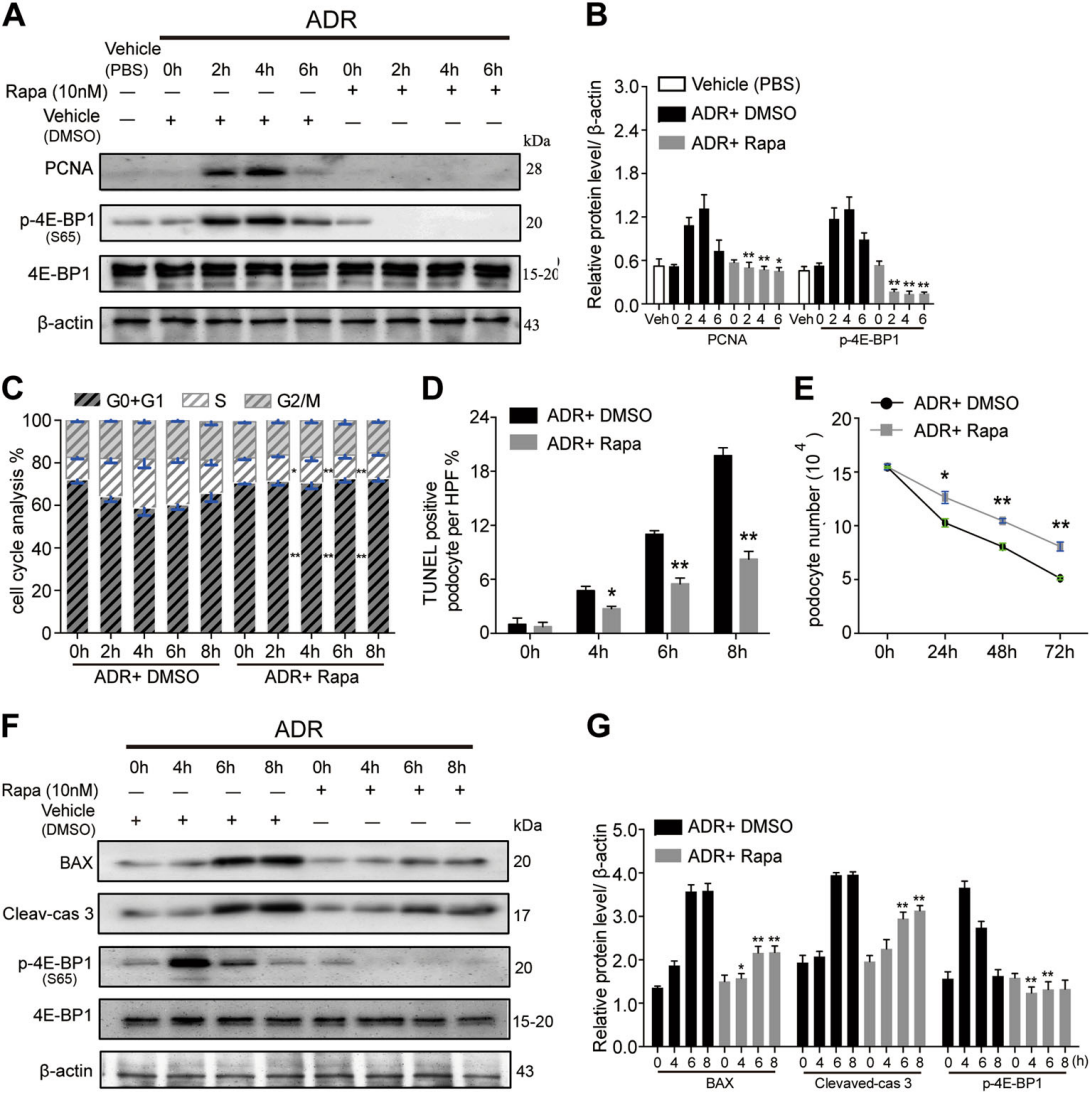
Blocking 4E-BP1 phosphorylation inhibited cell cycle re-entry and attenuated adriamycin-induced apoptosis in primary podocytes **a**, **b** Primary podocytes were treated with rapamycin (10 nm) or vehicle (DMSO) for 0, 2, 4, and 6 h respectively in presences of adriamycin (1 mg/L). Expression levels of total and the phosphorylated 4E-BP1 (S65), and PCNA were analyzed by immunoblotting. One representative blot is shown of at least three independent experiments **a**. **b** The statistic based on densitometric analysis of at least three independent experiments. Data were shown as means ± SEM ^**^P < 0.01 for ADR+Rapa vs. ADR+vehicle (DMSO). **c** Podocytes were incubated for 0, 2, 4, 6, or 8 h in the presence of adriamycin, with or without rapamycin treatment, and then FACS analysis was performed after propidium iodide staining. Quantitation of cell cycle phases from multiple (*n* ≥ 3), independent samples. Data were shown as means ± SEM, *P < 0.05, ^**^P < 0.01 for ADR+Rapa vs. ADR+vvehicle (DMSO). **d** TUNEL-positive rate was scored in adriamycin-treated podocytes with or without rapamycin addition for 0, 2, 4, 6, or 8 h, respectively. For each group, 45 randomly images was captured for calculation (15 images per slide, three slides per group). Data were shown as means ± SEM *P < 0.05, ^**^P < 0.01 for ADR+Rapa vs. ADR+vvehicle (DMSO). **e** Quantification of podocyte treated with vehicle (DMSO) or rapamycin in presence of adriamycin treatment for 0, 24, 46, or 72 h. Experiments were performed in triplicate and repeated 3 times with similar results. Data are presented as means ± SEM, *P < 0.05, ^**^P < 0.01 for ADR+Rapa vs. ADR+DMSO. **f**, **g** Immunoblotting analysis of BAX, cleaved-caspases-3, 4E-BP1, and p-4E-BP1 (S65) in rapamycin or DMSO-treated podocytes in presence of adriamycin treatment for 0, 4, 6, or 8 h. Shown were one from at least three separate experiments with similar results, the statistic based on densitometric analysis of 3 independent experiments. Data were shown as means ± SEM, *P < 0.05, **P < 0.01 for ADR+Rapa vs. ADR+vehicle (DMSO)

From the 4th week following adriamycin (1 mg/L) treatment, podocyte apoptosis was observed following cell cycle re-entry. As shown in Fig. 7, adriamycin-induced primary podocyte apoptosis from the 4th hour of treatment onward in a time-dependent manner. Prior to adriamycin treatment (0 h), a very low cleaved caspase-3-positive rate (1.5%) in podocytes was detected, which significantly increased to 7.5%, 28%, and 41.25% at 4, 6, and 8 h, respectively, following adriamycin treatment. Importantly, the adriamycin-induced increase of cleaved caspase-3 expression was significantly reduced by 4E-BP1 RNA interference (4.25%, 9.25%, and 14.5% at 4, 6, and 8 h, respectively) (Fig. 7a, b). Similarly, the protein expression level of cleaved caspase-3 and Bax, another key mediator of apoptosis, were notably elevated at the 6th and 8th hour of adriamycin treatment following podocyte cell cycle re-entry (Fig. 7e, f). In addition, cleaved caspase-3 and Bax protein expression increases were both diminished by 4E-BP1 RNA interference (Fig. 7e, f) or rapamycin treatment (Fig. 8f, g) in adriamycin-treated podocytes. The apoptosis rates of primary podocytes were confirmed using a terminal deoxynucleotidyl transferase dUTP nick end labeling (TUNEL) assay. And the TUNEL-positive rates in primary podocytes were elevated after 4, 6, and 8 h of adriamycin treatment (5.25%, 14.75%, and 30.25% by 4, 6, and 8 h, respectively) compared with the 0 h group (1%). Again, this increase was significantly reduced by 4E-BP1 RNA interference (3%, 6.25%, and 10% by 4, 6, and 8 h, respectively) (Fig. 7c) or pharmacologic rapamycin treatment (2.75%, 5.5%, and 8.25% by 4, 6, and 8 h, respectively) (Fig. 8d).

In order to verify the increased apoptotic cell death that resulted in decreased podocyte numbers, podocyte numbers were estimated at 24, 48, and 72 h following adriamycin treatment with or without 4E-BP1 siRNA treatment. Control siRNA-transfected podocytes treated with adriamycin showed a time-dependent decrease in podocyte numbers (9.2 × 10^4^, 7.9 × 10^4^, and 5.5 × 10^4^ by 24, 48, and 72 h, respectively). The podocyte loss was notably attenuated in 4E-BP1 siRNA-transfected podocytes treated with adriamycin (11.1 × 10^4^, 9.9 × 10^4^, and 9.2 × 10^4^ by 24, 48, and 72 h, respectively), indicating that 4E-BP1 RNA interference suppressed 4E-BP1 activity in adriamycin-treated podocytes and significantly prevented podocyte death (Fig. 7d). Similarly, the adriamycin-induced cell number decrease was markedly diminished by rapamycin (12.6 × 10^4^, 10.5 × 10^4^, and 8.1 × 10^4^ in rapamycin treated group by 24, 48, and 72 h, respectively) (Fig. 8e), confirming that inhibiting 4E-BP1 activation attenuated podocyte apoptotic cell death induced by adriamycin.

## Discussion

Adult podocytes are terminally differentiated epithelial cells that have exited from the cell cycle under normal conditions and do not typically proliferate following injury and loss. Nevertheless, forced re-entry of differentiated podocytes into the cell cycle is possible. Shankland et al.^34^ demonstrated that cytokine bFGF administration in rats with PHN promoted the occurrence of a limited DNA synthesis in rat podocytes. Using cell lines, Lasagni revealed that persistent activation of the Notch pathway induced podocyte cell cycle progression^35^. Moreover, biopsies of IgA nephropathy, FSGS, and Lupus nephritis showed bi- and poly-nucleated podocytes undergoing cell cycle re-entry^6^. However, objective evidence for podocyte cell cycle change under stress is still lacking.

The current study traced podocyte cell cycle changes both in adriamycin-induced glomerulonephritis mouse model built by *NPHS2 Cre; mT/mG* transgenic mice and in isolated primary podocytes treated with adriamycin. Differentiated podocytes re-entered into the cell cycle from the 1st to the 5th week after treatment in vivo. In vitro, adriamycin was able to stimulate primary podocyte cell cycle re-entry from the 2nd hour of treatment. This is a novel demonstration showing that in the early stage of adriamycin treatment, podocytes engaged the cell cycle both in vivo and in vitro. Importantly, cell cycle progression induced by adriamycin showed as a limited reaction, the proliferative indicator expression levels increased soon after adriamycin treatment, then diminished (although they remained above the basal level), indicating that the cell cycle re-entry is a reaction occurred soon after stimulation, but attenuated as long as injuries were aggravated.

Despite observing podocyte cell cycle re-entry, the molecular mechanisms regulating this change remain unclear. Till now, Laura demonstrated in a podocyte lineage that persistent activation of the Notch pathway induced podocytes to cross the G2/M checkpoint^35^. Marina reported that conditional overexpression of telomerase reverse transcriptase in the adult mouse kidney triggered a potent and reversible proliferative response in podocytes by activating the Wnt signaling pathway^36^. In this current study, by generating the *NPHS2; mT/mG* transgenic mice to build the adriamycin-induced glomerulonephritis model, we were able to investigate the molecular mechanism underlying podocyte cell cycle re-entry in vivo precisely. By isolating enhanced green fluorescent protein-labeled podocytes in vitro, the signaling pathway underlying the cell cycle progression in primary podocytes was investigated for the first time. Along with markedly elevated Ki67 and PCNA expression, notably enhanced phosphorylation of 4E-BP1 mediated by mTORC1 was detected from the 1st week in vivo or the 2nd hour in vitro following adriamycin treatment. Importantly, the p-4E-BP1 expression level showed exactly the same trend as Ki67 and PCNA both in vivo and in vitro, indicating that p-4E-BP1 could have a role in this podocyte cell cycle change induced by adriamycin.

It is well established that the effects of mTORC1 on cell proliferation in progenitor podocytes are mediated by 4E-BPs^27^. That is because a fundamental control point in the regulation of cell cycle initiation related protein synthesis is the formation of the eukaryotic initiation factor 4F (eIF–4F) complex. Activation of mTORC1 mediates the hyper-phosphorylation of 4E-BP1, consequently leading to its dissociation from eIF4E^30^. The released eIF4E binds to the 59-cap structure of mRNAs and, together with eIF4G and eIF4A, assembles the translation initiation complex eIF–4F, thus promoting the protein synthesis stimulated needed for cell proliferation^29^. In homeostasis, podocytes are fully differentiated and cannot proliferate, and mTORC1/4E-BP1 activity remains low to maintain normal podocyte function^2,21,23,37^. To prove whether this 4E-BP1 re-activation was implicated in cell cycle re-entry triggered by adriamycin, 4E-BP1 activity was inhibited by 4E-BP1 siRNA or rapamycin in primary podocytes treated with adriamycin. It was found that the increased Ki67-positive rate, the elevated PCNA expression level, and the improved S-phase fraction induced by adriamycin were all markedly diminished by 4E-BP1 RNA interference or pharmacologic rapamycin treatment. This novel finding demonstrated that 4E-BP1 re-activation positively mediated the cell cycle re-entry of differentiated podocytes induced by adriamycin.

Recently, several studies have suggested that aberrant mitosis of podocytes lead to podocyte loss, proteinuria, and subsequently, to the progression of glomerular diseases^17,20,38,39^. In the present study, apoptotic cell death was detected in podocytes following cell cycle re-entry. Given the demonstrated role of 4E-BP1 in mediating podocyte cell cycle re-entry, it would be interesting to test whether targeting 4E-BPs could suppress podocyte apoptotic cell death. It is noteworthy that the podocyte apoptosis was significantly attenuated by 4E-BP1 siRNA or rapamycin, indicating that cell cycle re-entry sensitized podocytes to adriamycin-induced apoptosis. Therefore, 4E-BP1 could be used as a regulator to manipulate podocyte cell cycle re-entry, preventing podocyte injury or death and thereby providing a novel strategy for alleviating glomerular diseases.

Podocyte cell cycle re-entry leads to podocyte injury and loss^6,10,17,20,35,39,40^. The resulting loss of glomerular barrier function allows for massive proteinuria, sclerosis, and adhesions of the glomerular tuft to Bowman’s capsule in FSGS patients^22,41–44^. Now for patients with steroid-resistant FSGS, the mTORC1/4E-BP1-signaling inhibitor, rapamycin, also known as sirolimus, has been used to ameliorate FSGS progression through antiproliferation, suppression of podocyte immune responses, and activation of autophagy^45–47^. Accordingly, we are speculating that, using sirolimus therapy to inhibit 4E-BP1 phosphorylation and cell cycle re-entry as early as the phosphorylated 4E-BP1 and Ki67 was detected in podocyte in minimal change disease (MCD) patients (Figure S5, Figure S6), might be a useful approach to diminish podocyte apoptosis and loss in the initial phases of glomerular injury and thus prevent the formation of sclerotic lesions.

In conclusion, the results of this study demonstrated that 4E-BP1 activation regulated the amount of cell cycle re-entry provided by differentiated podocytes and the degree of podocyte apoptosis, opening the previously unthinkable possibility that in patients with glomerular disorders 4E-BP1 activity might be manipulated to ameliorate podocyte injury.

## Materials and methods

### Reagents

Adriamycin, Type IV collagenase Rapamycin, and DNase were all purchased from Sigma (China-Mainland), DMEM (Dulbecco’s modified Eagle’s medium) low-glucose medium was from Biological Industries, Israel. Cell strainers were purchased from Thermo Fisher Scientific (China) Co., Ltd. Anti-nephrin antibody was purchased from R&D Systems, Inc. (Minneapolis), anti-podocin antibody was from Proteintech Group, Inc. (China), anti-WT1 antibody was purchased from Santa Cruz Biotechnology, Inc. (California), anti-Ki67 antibody, and anti-PCNA antibody were purchased from Abcam plc. (Shanghai-China), anti-Ser65-phosphorylated 4E-BP1, and anti-cleaved caspase-3 was purchased from Cell Signaling Technology, Inc.(MA). DyLight 488- and Alexa Fluor™ 594- conjugated secondary antibodies were both purchased from Thermo Fisher Scientific Inc. Tunnel assay kit (In Situ Cell Death Detection Kit, Fluorescein) was purchased from Roche Ltd (Shanghai-China).

### Mice experiments

Permissions on performing animal experiments for research purposes (No. 20150713) was approved by the Ethical Committees of School of Basic Medical Sciences, Fudan University. All procedures were carried out according to the approved guidelines. Mice were purchased from the Jackson Laboratories and housed in our facilities providing free access to water and regular chew. To generate *NPHS2 Cre; mT/mG* mice, *mT/mG* (*Gt(ROSA)26Sor*^*tm4(ACTB-tdTomato,-EGFP)Luo*^) mice was crossed to NPHS2 cre mice. Mice were maintained on the mixed (B6.129SvJ × C57BL/6J) genetic background. For the inducement of podocyte injury, 8-week-old male mice received a single dose of adriamycin (15 mg/kilogram (kg) body weight) injection via the tail vein.

### Original fluorescent images from *NPHS2 Cre; mT/mG* mice

Kidney tissues from *NPHS2 Cre; mT/mG* mice were fixed by paraformaldehyde and embedded by paraffin, always be kept in dark and avoided keeping in 70% ethanol for > 6 h. After deparaffinization, the slides were hydrated by dropping in 100% ethanol, 95% ethanol, 85% ethanol, and 70% ethanol solutions, respectively, followed by rinsing in PBS for 5 min, counterstaining with 4′,6-diamidino-2-phenylindole (DAPI) and mounted by mounting medium. The images were captured using Vectra Imaging System (Caliper Life Sciences, Inc., MA, USA).

### Immunofluorescence staining of tissue sections

The paraformaldehyde-fixed and paraffin-embedded sections were dewaxed and hydrated. The antigen was retrieved in citrate buffer. The sections were blocked with 2% normal goat serum, incubated with primary antibodies at the following dilutions: Ki67 (1:100), WT1(1:100), Ser (37, 46)-phosphorylated 4E-BP1 (1:100), podocin (1:100), nephrin (1:100), overnight at 4 °C, and then incubated with DyLight 488- and Alexa Fluor™ 594- conjugated secondary antibodies at 37 °C for 1 h. Nuclei were counterstained with DAPI (1:1000; Sigma-Aldrich). Images were captured by Vectra Imaging System as above. Podocin or p-4E-BP1 (S37, 46) positive area in glomeruli were measured by using CellSens Entry software, Olympus.

### Immunofluorescence staining of podocytes grown on the glass slides

The cells slides in which primary podocytes express fluorescence protein mG were fixed with 4% paraformadelhyde for 30 min at room temperature. After rinsing twice with PBS, cells slides were permeabilized with 0.2% Triton X-100 in PBS for 10 min, blocked with 2% goat serum in PBS for 1 h, incubated with primary antibody (Ki67; Ser (37, 46)-phosphorylated 4E-BP1; cleaved caspase-3; WT1; nephrin) diluted as 1:100 at 4 °C overnight and secondary antibodies at room temperature for 1 h, and counterstained with DAPI. After rinsing three times with PBS, cells slides were mounted with Prolong Gold Antifade Reagent (Thermo Fisher Scientific Inc.). Images were taken using Vectra Imaging System as above.

### Electron microscopy

Kidney tissues from mice were fixed in 2.5% glutaraldehyde overnight and processed for standard SEM at Center of Pathological Imaging, School of Basic Medical Sciences, Fudan University. We took eight randomly unbiased electron microscopic photographs of glomeruli at magnifications of × 6000 in each case.

### Urine albumin excretion, proteinuria, BUN, and creatinine analysis

Urine and blood samples were both collected weekly from mice for 5 weeks after adriamycin or vehicle injection, mice urine albumin concentration and urine creatinine concentration was measured using the assay kit from Nanjing Jiancheng Bioengineering Institute, China. Excretion of albumin is expressed as urine albumin to creatinine ratio. In addition, urine albumin concentration was confirmed using SDS–PAGE and coomassie blue staining, 5 μl urine per lane. Measurements of 1 and 2 μg of bovine serum albumin were used as controls to show the location of albumin. BUN and plasma creatinine levels were immediately measured using Liquid Urea Nitrogen Reagent Set and Creatinine Assay kit (Nanjing Jiancheng Bioengineering Institute, China) according to the manufacturer’s protocol.

### Podocyte isolation (for in vivo experiment)

Kidneys from experimental mice were decapsulated, excised and minced, then passed through a system of sieves with decreasing pore diameters (250, 150, and 100 μm) to obtain a suspension of glomerular in PBS. Then the glomeruli were digested with 1 mg/ml of type IV collagenase containing 5000 U/ml DNase at 37 °C for 2 h. After that, the suspension of glomerular cells was filtered through the 40 μm sieve. The mG-labeled podocytes were isolated and directly analyzed by FACS.

### Primary podocyte culture and treatment

Male *NPHS2 Cre; mT/mG* mice at 5–6 weeks of age was used for primary podocyte culture. The glomerular cells suspension obtained as above was plated in type I collagen-coated culture flasks, cultured in DMEM low-glucose supplemented with 10% FBS, Insulin, Transferrin, Sodium Selenite Media Supplement, ITS (Sigma-Aldrich), Hydrocortisone, Na-3,3′, 5-Ttiiodo-L-Thyronine,100 U/ml penicillin, and 100 μg/ml streptomycin, and incubated at 37 °C in 95% air 5% CO2 for at least 15 days. Then the outgrowing cells were trypsinized and mG-labeled podocyte was isolated through FACS. And then the pure podocytes were seeded in culture flasks, and incubated at 37 °C in 95% air 5% CO2 for 3 days. Cell phenotypes were determined by using podocyte-specific antibodies against WT1 and nephrin. Primary podocytes were treated with adriamycin at 1 mg/L for 0, 2, 4, 6, 8, 24, 48, and 72 h, respectively.

### Protein isolation and immunoblotting analysis

The harvest of the pellet of podocytes using cell lysis buffer was as described before lysates (40 μg), measured by BCA Protein Assay Reagent kit (Piece, IL, USA) were used for immunoblotting analysis^48^. The podocytes were isolated from adriamycin-treated mice as described above, and then lysed in the lysis buffer in the presence of protease inhibitor cocktail and followed by centrifugation. The lysate measurement and the immunoblotting were both performed as described previously^49^. The optical density of the bands, normalized to β-actin, was determined by Chemi-Doc Imaging System (Bio-Rad).

### Transfection of small interfering RNA (siRNA) against 4E-BP1

Primary podocytes were seeded in 12-well plates at 2.0 × 10^5^ per well and the cells were transfected with siRNA against 4E-BP1 (si-4E-BP1) or a control siRNA (Gene Pharma co.ltd, China) at a final siRNA concentration of 25 nM by using TransIT-X2® Dynamic Delivery System (Mirus Bio LLC. U.S.). After 48 h of incubation, the cells were treated with adriamycin at 1 mg/L for 4, 6, or 8 h.

### Cell counting for primary podocytes

Primary podocytes cultured in 12-well plates were trypsinized in 0.5 ml of trypsin solution and suspended in 1 ml of complete media each well. For quantification by cell counting, primary podocytes were taken from 12-well plates after cultures had been uniformly resuspended by mixing using a 3 ml transfer pipette (BD Falcon). Ten microliters of the homogeneous cell samples were mixed at a 1:1 ratio with Trypan Blue (Sigma-Aldrich), then 10 µl of the cell-Trypan Blue mixture was loaded on a hemocytometer (Qiujing, Shanghai) and visually examined under an inverted light microscope (Olympus) and counted using trypan blue exclusion to distinguish between live and dead cells. Counts from two 1 × 1 mm quadrants were averaged and multiplied by 1 × 10^4^ × dilution factor to obtain number of cells/ml.

### Cell cycle analysis

For cell cycle analysis, podocytes were harvested after 0, 2, 4, 6, and 8 h of treatment. After detaching, the cells were washed with PBS, and centrifuged at 200 × *g* for 5 min. The cell pallet was resuspended in propidium iodide solution and incubated for 15 min at room temperature. Then analyzed by a flow cytometer (FACS Calibur, Becton Dickinson, USA). Using 488 nm excitation and a filter >600 nm for PI detection, and the cell cycle phase quantitation was performed by CELLQuest software.

### Renal biopsies

Renal needle biopsies were collected from the nephrology laboratory, Department of Pathology, School of Basic Medical Sciences, Fudan University. The analyzed kidney biopsies were from adult (42 ± 20 years) patients with a diagnosis of MCD or FSGS by immunofluorescence staining, hematoxylin and eosin staining and electron microscopy. All patients exhibited nephritic range proteinuria at the time of biopsy. The normal kidney biopsies were from the paracarcinoma tissue. The histologic analyses of the present study were approved by the Ethical Committees of School of Basic Medical Sciences, Fudan University (2017-Y009).

### Statistical analyses

Statistical analysis was performed using Prism (Graph Pad Software) and SPSS V20.0 (IBM Corp.). The experiments were all repeated for at least three times in independent biological replicates. The Kolmogorov–Smirnov test was used to verify the normal distribution of variables. Exploratory comparison of normally distributed and non-normally distributed independent groups was performed using *t* tests and Mann–Whitney *U* test . A two tailed *P* value ≤ 0.05 was considered statistically significant.

## Supplementary information

Supplemental Figure legends

Supplemental Figure 1

Supplemental Figure 2

Supplemental Figure 3

Supplemental Figure 4

Supplemental Figure 5

Supplemental Figure 6
