# Supplementary material for: Inhibiting 4E-BP1 re-activation represses podocyte cell cycle re-entry and apoptosis induced by adriamycin
Source: Cell Death Dis. 2019 Mar 11;10(3):241. doi: 10.1038/s41419-019-1480-x (PMC6411872; doi:10.1038/s41419-019-1480-x)
Supplement: Supplementary file 1 — Supplemental Figure legends [file 41419_2019_1480_MOESM1_ESM.docx]

**Supplementary Figure 1. Generating podocyte-specific membrane- labeled EGFP transgenic mice.** (A) A schematic depicting the generation of podocyte-specific membrane-localized enhanced green fluorescence protein (EGFP) transgenic mice (*NPHS2 Cre; mT/mG* mice). (B) Fluorescence labeling pattern in the glomerulus showed podocyte was membrane-labeled with EGFP (mG) and other cells with red fluorescence protein td-Tomato (mT). Shown are representative kidney sections with similar results.

**Supplementary Figure 2. Podocyte injury was observed from the 4th week after adriamycin** **treatment.** (A) Urinary albumin/ creatinine ratios of adriamycin or vehicle treated mice. (B) Urinary albumin of experimental mice (representative SDS-polyacrylamide gel coomassie blue staining). (C&D) BUN and plasma creatinine level of adriamycin or vehicle treated mice. Datas were expressed as means ± SEM (n=4 for each group). **P<0.05, **P<0.01* for adriamycin treated mice *vs.* vehicle treated mice.

**Supplementary Figure 3. Adriamycin stimulated podocyte cell cycle re-entry.** (A) Double immunofluorescence staining with podocin and Ki67 revealed an increase of Ki67 positive podocytes (yellow arrow) per glomerulus in adriamycin treated mice compared to the vehicle group. (B) The number of Ki67-positive podocytes was calculated from 40 randomly captured images for each group (10 images per mouse, 4 mice per group) at each time point. Only Ki67-positive nuclei within podocin-positive area were counted for the numerator, which was divided by the sum of glomeruli within captured images, showing an increase of podocin and Ki67 double positive cells in the first two weeks and then gradually decreased from the 3rd week but still remained higher than control group until tne 5th week after adriamycin treatment. Datas were presented as means ± SEM, ***P<0.01* for adriamycin treated mice *vs.* vehicle treated mice. (Scale bars: 100 μm)

**Supplementary Figure 4. Culturing primary podocytes isolated from *NPHS2 Cre; mT/ mG* mice.** (A) Glomeruli were isolated from *NPHS2 Cre; mT/ mG* mice and cultured in cell culture medium, showing a bulk of podocytes (membrane labeled with EGFP) growing out and surrounding the central glomeruli (yellow arrow). (B) Culturing pure m-EGFP labeled podocytes obtained through FACS. (C&D) Immunofluorescence staining with WT1 (C) or nephrin (D) to identify the EGFP labeled podocytes. Shown are representative images from 1 of 3 separate experiments with similar results.

**Supplementary Figure 5. Ki67 positive podocytes were detected in the human renal biopsies of MCD patients.** (A) Representative kidney sections with H&E staining from normal controls (paracancerous tissue), MCD and FSGS patients. (B) Double immunofluorescence staining of nephrin and Ki67 for renal biopsies from 4 cases of normal kidney tissues, 20 cases of MCD, and 25 cases of FSGS. Shown were representative images with similar results. (C) After eluting the antibodies in the same section of (B), double immunofluorescence staining with Ki67 and WT1 revealed that the Ki67 positive cell found in MCD was also positive for WT1, confirming the Ki67 positive cell is podocyte. (Scale bars: 100 μm)

**Supplementary Figure 6. P-4E-BP1 positive podocytes were detected in the human renal biopsies of MCD patients.** (A) Double immunofluorescence staining of nephrin and p-4E-BP1 (S37/ 46) for renal biopsies from 4 cases of normal kidney tissues and 8 cases of MCD patients, and p-4E-BP1 (S37/ 46) positive podocyte were detected in one MCD patient. Shown were representative images with similar results. (Scale bars: 100 μm)
