# Supplementary figures and images for: Inhibiting 4E-BP1 re-activation represses podocyte cell cycle re-entry and apoptosis induced by adriamycin
Source: Cell Death Dis. 2019 Mar 11;10(3):241. doi: 10.1038/s41419-019-1480-x (PMC6411872; doi:10.1038/s41419-019-1480-x)

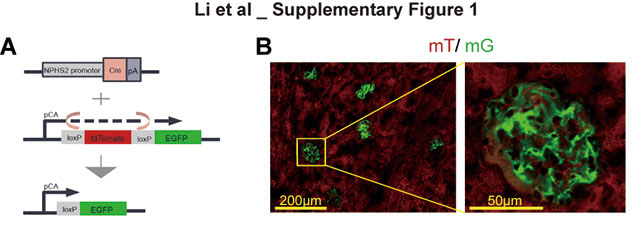

Supplement: Supplementary file 2 — Supplemental Figure 1 [file 41419_2019_1480_MOESM2_ESM.jpg]

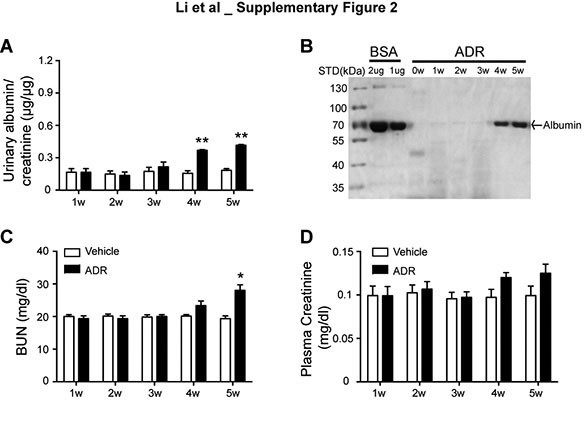

Supplement: Supplementary file 3 — Supplemental Figure 2 [file 41419_2019_1480_MOESM3_ESM.jpg]

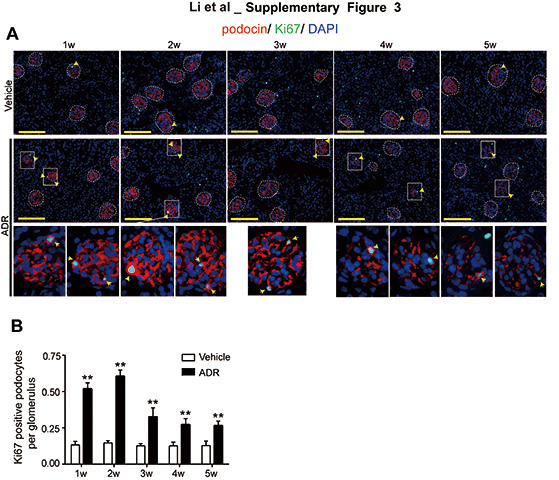

Supplement: Supplementary file 4 — Supplemental Figure 3 [file 41419_2019_1480_MOESM4_ESM.tif]

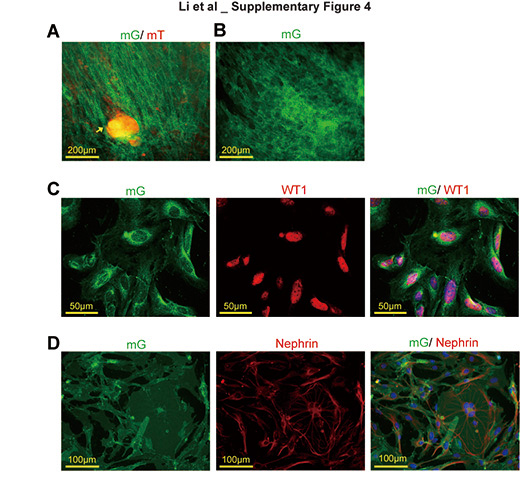

Supplement: Supplementary file 5 — Supplemental Figure 4 [file 41419_2019_1480_MOESM5_ESM.tif]

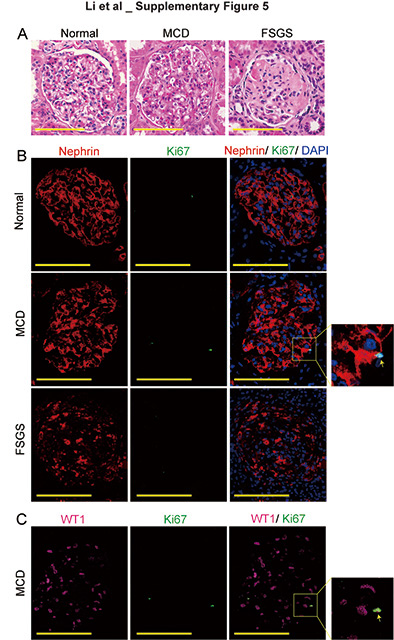

Supplement: Supplementary file 6 — Supplemental Figure 5 [file 41419_2019_1480_MOESM6_ESM.tif]

Li et al \_ Supplementary Figure 6

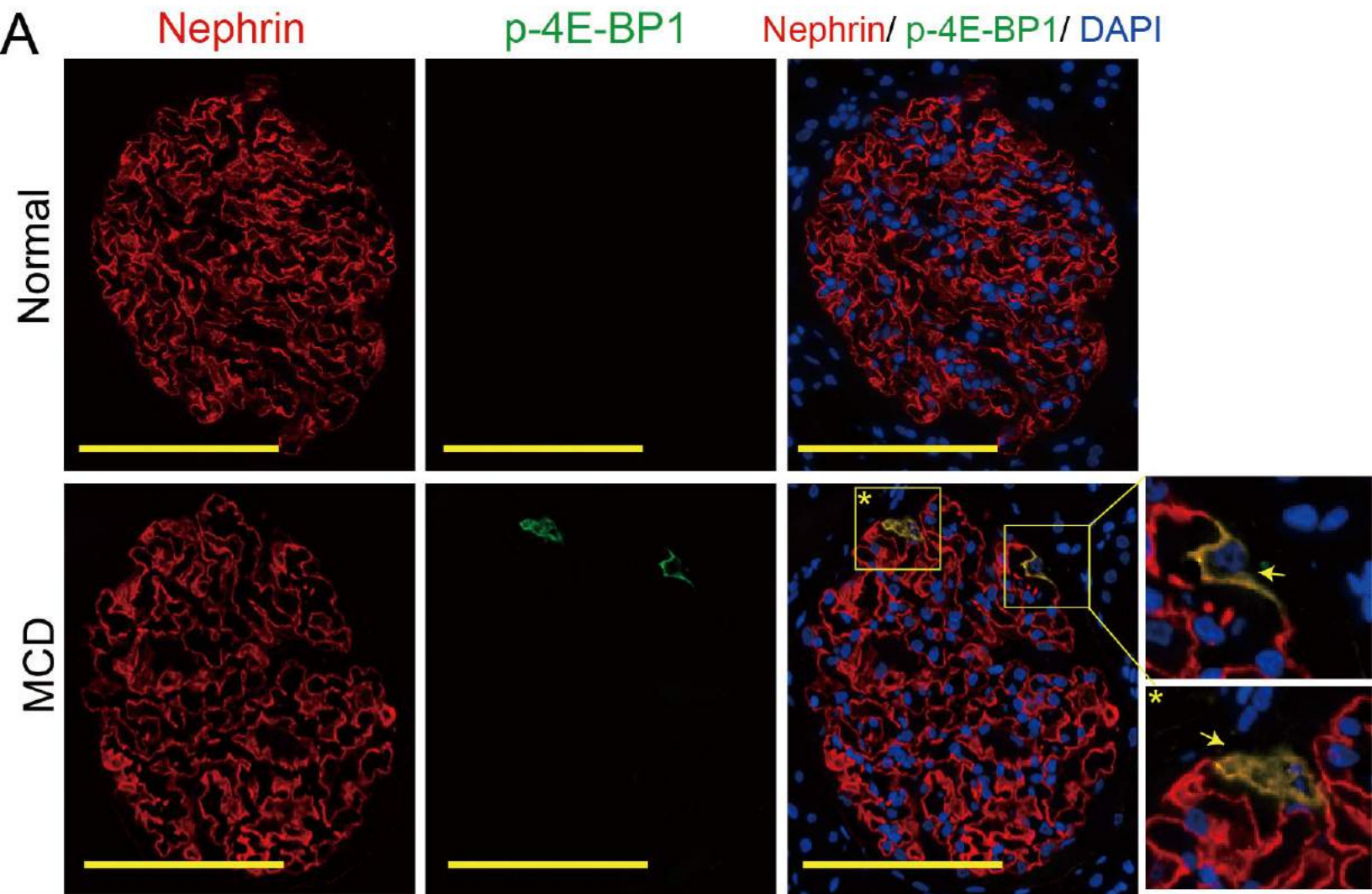

Supplement: Supplementary file 7 — Supplemental Figure 6 [file 41419_2019_1480_MOESM7_ESM.pdf]
